# Sequence Composition and Gene Content of the Short Arm of Rye (*Secale cereale*) Chromosome 1

**DOI:** 10.1371/journal.pone.0030784

**Published:** 2012-02-06

**Authors:** Silvia Fluch, Dieter Kopecky, Kornel Burg, Hana Šimková, Stefan Taudien, Andreas Petzold, Marie Kubaláková, Matthias Platzer, Maria Berenyi, Siegfried Krainer, Jaroslav Doležel, Tamas Lelley

**Affiliations:** 1 Health and Environment Department, Bioresources, Austrian Institute of Technology (AIT), Tulln, Austria; 2 Centre of the Region Haná for Biotechnological and Agricultural Research, Institute of Experimental Botany, Olomouc, Czech Republic; 3 Leibniz Institute for Age Research (Fritz Lipmann Institute), Jena, Germany; 4 Department of Agrobiotechnology, Institute for Biotechnology in Plant Production (IFA-Tulln), University of Natural Resources and Applied Life Sciences (BOKU), Vienna, Austria; University of Lausanne, Switzerland

## Abstract

**Background:**

The purpose of the study is to elucidate the sequence composition of the short arm of rye chromosome 1 (*Secale cereale*) with special focus on its gene content, because this portion of the rye genome is an integrated part of several hundreds of bread wheat varieties worldwide.

**Methodology/Principal Findings:**

Multiple Displacement Amplification of 1RS DNA, obtained from flow sorted 1RS chromosomes, using 1RS ditelosomic wheat-rye addition line, and subsequent Roche 454FLX sequencing of this DNA yielded 195,313,589 bp sequence information. This quantity of sequence information resulted in 0.43× sequence coverage of the 1RS chromosome arm, permitting the identification of genes with estimated probability of 95%. A detailed analysis revealed that more than 5% of the 1RS sequence consisted of gene space, identifying at least 3,121 gene loci representing 1,882 different gene functions. Repetitive elements comprised about 72% of the 1RS sequence, Gypsy/Sabrina (13.3%) being the most abundant. More than four thousand simple sequence repeat (SSR) sites mostly located in gene related sequence reads were identified for possible marker development. The existence of chloroplast insertions in 1RS has been verified by identifying chimeric chloroplast-genomic sequence reads. Synteny analysis of 1RS to the full genomes of *Oryza sativa* and *Brachypodium distachyon* revealed that about half of the genes of 1RS correspond to the distal end of the short arm of rice chromosome 5 and the proximal region of the long arm of *Brachypodium distachyon* chromosome 2. Comparison of the gene content of 1RS to 1HS barley chromosome arm revealed high conservation of genes related to chromosome 5 of rice.

**Conclusions:**

The present study revealed the gene content and potential gene functions on this chromosome arm and demonstrated numerous sequence elements like SSRs and gene-related sequences, which can be utilised for future research as well as in breeding of wheat and rye.

## Introduction

From the beginning of the last century, several attempts have been made to integrate useful genetic variations of related species into cultivated hexaploid wheat by means of inter-specific hybridisation. In the 1930s in Germany, hybridisation between a cultivar of bread wheat (*Triticum aestivum* L.) and cv. ‘Petkus’ of rye (*Secale cereale* L.) was followed by a spontaneous homoeologous substitution, replacing wheat chromosome 1B by chromosome 1R of rye. The rye chromosome fully compensated for the missing chromosome 1B. However, its presence remained undetected until the early 1970s [1]. Later it was found that chromosome 1R could largely compensate also for the absence of chromosomes 1A and 1D belonging to the same homoeologous group.

Subsequent crossings of hexaploid wheat lines bearing the 1B/1R substitution with lines without the substitution chromosome led to the emergence of the translocation chromosome 1BL.1RS, in which the short arm of 1R (1RS) was translocated to the long arm of the wheat chromosome 1B (1BL), replacing the 1BS short arm of wheat. Later, translocations involving the chromosome arms 1AL and 1DL were also developed [2], [3]. All of these translocations were found to confer resistance to several diseases caused by pathogens in wheat, including powdery mildew, leaf rust, stem rust, yellow rust and insects, as well as Russian wheat aphid, green bug and wheat curl mite, the last being the vector of the wheat streak mosaic virus [2], [4]–[9]. Importantly, in certain wheat backgrounds the 1RS chromosome arm was found to improve adaptation to low moisture conditions and increase the yield of wheat [10], [11]. Recently it has been suggested that the yield increase may be due to the enlarged root biomass [12]. A corresponding QTL region was localised in the distal part of 1RS [13], [14].

Due to these valuable characteristics, wheat lines carrying 1RS have been integrated into CIMMYT's (Centro Internacional de Mejoramiento de Maíz y Trigo; International Maize and Wheat Improvement Center) wheat breeding program. Through this program, chromosome 1RS was distributed worldwide. Hundreds of wheat cultivars were later found carrying the 1BL.1RS translocation [15], [16]. In the US, wheat varieties with a 1AL.1RS translocation were developed carrying 1RS from the rye cultivar Insave. The distribution of this translocation, however, remained confined to the US [7]. Wheat varieties with 1DL.1RS chromosome have never gained practical relevance.

Rye is a close relative of wheat. Its 1C-value was estimated to be 8.095 pg DNA [17] or 7917 Mbp [18]. Based on the measurement of chromosome lengths by Schlegel et al. [19], the molecular size of the short arm of 1R is estimated to be 442 Mbp. Similar to other grass species, the rye genome contains large quantities of non-coding repetitive DNA, reported to comprise 84–92% of the genome [20], [21]. The short arm of 1R carries a satellite separated by a secondary constriction, which is also known as the nucleolus-organizing region (NOR) and contains genes coding for 45S rRNA. The number of the 45S rDNA genes was estimated to be about 2,000, amounting to about 3% of 1RS DNA [22]. The satellite also bears a 5S-rDNA locus with about 5,000 copies of the 5S rDNA gene, constituting 0.4% of 1RS DNA [22]. Genetic and physical mapping of 1RS showed that most of the previously identified genes cluster within the satellite of 1RS [23]–[31]. In this region, secalin genes [32] as well as a number of genes conferring resistance to leaf and stripe rust [8] Russian wheat aphid [9], green bug and wheat curl mite [7] were localised. Several molecular markers having been developed for 1RS are also located in the distal region [27], [31], which is marked by close synteny to the short arm of the homoeologous group 1 of wheat [33].

The colinearity of plant genomes has been established by the development of genetic markers and linkage mapping. Colinearity of genomes has been characterised in detail for grass species [34], [35]. Detailed colinearity maps of grasses have culminated in DNA sequence-based comparisons [36]. The availability of nearly complete genome sequences in small genome cereals such as rice, sorghum and *Brachypodium distachyon* facilitates colinearity analysis of large genome cereals using these small genomes as templates. Recently, Hackauf et al. [37] assessed colinearity between rye and rice, using 334 genetically mapped rye EST-based markers. They identified corresponding regions of the rice genome by in silico correlation of rye to rice genes. In agreement with previous observations [38] and based on gene sequences, Hackauf et al. [37] established a statistically significant colinearity of rice chromosome 5 and rye chromosome 1.

Recent progress in dissecting large cereal genomes to smaller parts by flow-sorting single chromosomes and chromosome arms, and the possibility of obtaining sufficient quantities of DNA from as few as 10,000 chromosomes by multiple displacement amplification (MDA), have greatly facilitated colinearity analysis in cereals with large genomes [39]–[43]. Flow cytometric chromosome sorting for rye was developed by Kubaláková et al. [44] and Šimková et al. [45]; the authors used flow cytometry to sort the chromosome arm 1RS for construction of a 1RS-specific BAC library. The availability of a 1RS-specific BAC library permitted the first analysis of the sequence composition of the rye genome, and 1RS in particular, by sequencing BAC ends [21]. Simultaneously, Kofler et al. [46] developed a set of SSR markers from the DNA of flow-sorted 1RS. Despite these efforts, sequence data concerning the rye genome (9240 sequence entries, amounting to 5557 unigenes) and knowledge of its colinearity with other grasses are still very limited. Thus, the development of markers linked with traits of interest and positional gene cloning are also restricted. Reducing the complexity of whole genomes by isolating single chromosomes and sequencing them by next-generation sequencing technology is an attractive means of generating large quantities of sequence data from specific genome regions to identify gene sequences and other loci that may serve as markers, and to establish colinearity with other species as it was demonstrated for barley [41], [42] and wheat [43].

The present study deals with the sequence composition analysis of the chromosome arm 1RS. Shotgun 454 pyrosequencing of DNA obtained from flow-sorted 1RS resulted in 0.43× coverage of the chromosome arm's sequence. This novel approach also permitted a detailed description of the gene space as well as the repetitive portion of this important chromosome arm including SSR regions, which may be used as first hand genetic markers. Comparative gene content analysis of 1RS genes with those of rice, *Brachypodium distachyon* and the short arm of the barley 1H chromosome was established, providing a basis for the analysis of changes in genome structure accompanying the evolution of cultivated grasses.

## Results

### The dataset and its quality

**Figure 1:**
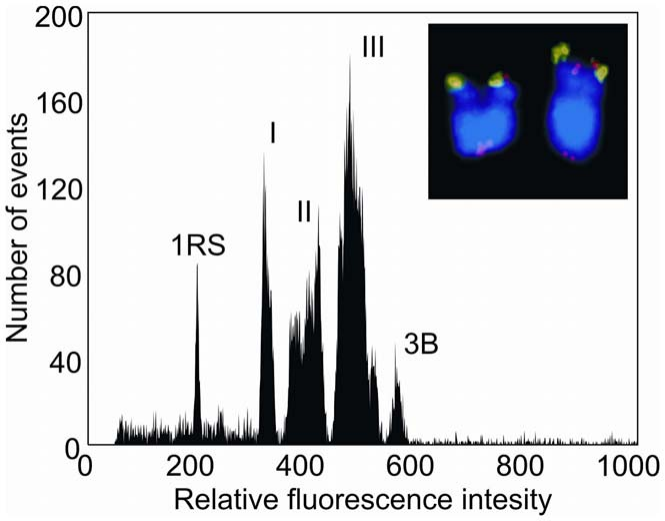
Histogram of relative fluorescence intensity (‘flow karyotype’) obtained after flow cytometric analysis of DAPI-stained chromosome suspension of wheat-rye 1RS telosome addition line. The karyotype contains four peaks representing the chromosomes of wheat (labelled I, II, III, and 3B) and a peak of the telocentric chromosome 1RS. The peak of chromosome 1RS is clearly discernible and chromosomes can be easily sorted. Insert: Images of the flow-sorted chromosome 1RS after FISH with probes for telomeric sequences (red) and pSc200 (green) subtelomeric DNA sequences. The chromosomes were counterstained with DAPI (blue).

The telocentric chromosome 1RS is not stable inherited in wheat-rye addition lines. Therefore, cytological control of the seeds used for multiplication was required in order to ensure the presence of a sufficient number of 1RS in the samples for flow cytometry. This resulted in good resolution of the 1RS arm on histograms of chromosome fluorescence intensity (flow karyotypes), high yields during the chromosome sorting, and low contamination of sorted fractions by other chromosomes. The flow karyotype consisted of four peaks representing various wheat chromosomes and a well resolved peak of chromosome 1RS (Figure 1). The telosome could easily be sorted and the sorted 1RS were identified by FISH with probes representing subtelomeric heterochromatin (pSc200) and telomeric repeats (Figure 1). The purity of samples in the sorted fraction varied between 89 and 93%. The purest sample was used for further processing. This sample comprised 93% of 1RS and 7% of various wheat chromosomes and their fragments. After DNA purification, 14 ng of chromosomal DNA was obtained from 30,000 flow-sorted 1RS telosomes, which were subsequently used for multiple displacement amplification (MDA), essentially as described by Simková et al. [40]. The yield of amplified 1RS DNA was 5.1 µg.

#### The sequence reads

Roche 454 FLX sequencing of 1RS DNA resulted in 942,768 sequence reads. Within this dataset 42,167 sequence reads (4.5%) were identified as “identical sequence”, i.e. were found at least twice in the dataset, and were considered as putative artefacts most likely generated by emulsion PCR [47]. Leaving one sequence read in the dataset, the additional copies were removed along with reads shorter than 50 bp. The remaining 895,199 sequence reads of the dataset constituted 195,313,589 bp sequence information with an average sequence length of 219 bp, and were used for further analysis (Table S1). The putative size of the 1RS chromosome, which comprises about 5.6% of the total rye genome [19], is about 442 Mbp. Consequently the available sequence information equals approximately 43.2% of the 1RS chromosome arm sequence, not considering possible overlaps.

### Sequence composition of 1RS

#### More than 5% of all sequence reads are related to the gene space of 1RS

To identify putative gene related sequence reads eight sequence databases were used (Table S2). Separate databases were used for identification of 5S and 45S rDNA as well as the secalin genes. Using a stepwise procedure as outlined in Material and Methods we identified 5.45% of all reads representing gene space (48,841 reads). Of these 2.05% (18,325 reads) represented the two ribosomal loci (1.78% and 0.26% for 45S and 5S rDNA loci, respectively) and 0.05% the secalin loci (400 reads) (Table S1).

#### Ribosomal loci of 1RS

The 45S and the 5S rDNA loci are found in close vicinity to the distal portion of the chromosome arm as shown by *in situ* hybridisation, and were reported to be present in about 2,000 and 5,000 copies [22] comprising about 18 Mbp (3,9%) and 2.3 Mbp (0,5%) of the 1RS genome, respectively. This suggested a 7.8-fold difference in the spatial requirement of the two loci, which is also reflected in our results: we identified 15,962 sequence reads (1.78%) for the 45S rDNA and 2,361 sequence reads (0.26%) for the 5S rDNA locus, yielding a 6.75-fold difference (Table S1).

#### Secalin Sec-1 locus

Three secalin loci have been reported for rye. *Sec-1* and *Sec-3* loci are located on the short and the long arm of chromosome 1R, respectively. The *Sec-1* locus on 1RS harbours the γ-secalin 40 kD and ω-secalin (∼45 kD) genes while *Sec-3* on 1RL contains the HMW-secalin locus (∼100 kD). The *Sec-2* locus found on the short arm of chromosome 2R codes for the 75 kD γ-secalin. In all 400 sequence reads (0.05%) were found to be related to the secalin genes. The majority - 316 of the identified reads - represented ω-secalin genes, which were reported to be present in about 15 copies in 1RS [48] while 45 of them were related to γ-secalin. In addition 39 sequence reads could not be assigned to γ-secalin or ω-secalin genes because of the high level of sequence homology of the two secalins in certain regions (Table S1).

#### Annotation of sequence reads reveals at least 3,121 gene loci and at least 1,882 different gene functions on 1RS

Excluding the ribosomal and secalin genes 3.36% of all sequence reads (30,118 reads) identified the gene space of 1RS. Based on homology to the rice genome, these sequence reads correspond to 3,121 different rice gene models (Table 1). An additional 3,638 putative gene loci, which had no homology to the RGA (Rice Genome Annotation) set of gene models were identified on the basis of the entries in the NCBI UniGene (*Hordeum*, *Oryza* and *Triticum*), RAP-DB, Rye UniGene, Wheat ABDS and NT Cereal databases), respectively, which had no homology to the RGA (Rice Genome Annotation) set of gene models. The estimated number of 3,638 must be treated with caution. Even when working with unigene sets containing just one entry/gene within a given set, sequences representing the same gene may exist in between the unigene datasets. Therefore, these results suggest the presence of at least 3,121 but up to 6,759 gene loci on 1RS, including the two secalin loci as well, and not considering the ribosomal genes (Table 1 and details in Table S3).

**Table 1:** Number of gene loci and gene functions identified in 1RS.

Gene loci identified on 1RS	No. of loci
RGA related loci	3121
Loci with no RGA annotation	3636
Secalins	2
**Maximum number of putative loci on 1RS**	**6759**
**Gene functions identified on 1RS**	
Secalins	2
RGA based annotation	1608
Other DBs based annotation*	272
**Functionally annotated genes**	**1882**
Expressed genes**	3776
Hypothetical genes***	76
**Maximum number of putative functions**	**5732**

*RAP-DB, Triticum/Hordeum/Oryza Unigene DB, Secale Unigene DB, Wheat 1ABDS DB, Brachypodium distachyon Protein DB and NT Cereal DB.

**All EST entries with no annotation.

***Based on the RGA database.

Identifying the number of gene functions on 1RS was based on the available rice dataset in the RGA database. This set was supplemented with functional annotations found in the *Triticum*, *Hordeum* UniGene and NT Cereal databases. Using these two approaches, we identified 1,882 gene functions, including γ and ω secalins. Of these 1,608 were based on the rice RGA dataset while 272 functions were recovered by the *Triticum* UniGene, *Hordeum* UniGene and NT Cereal databases (Table 1). Furthermore a maximum of 3,852 additional gene functions may be hypothesised based on either existing but functionally not annotated EST sequences (expressed genes), or in silico identified putative gene regions (hypothetical genes). Functional gene annotation revealed 43 *disease resistance* loci assignable to 12 functional categories, as well as 29 loci of 10 functional categories related to *Powdery mildew resistance* (Table S3).

#### Genes present on 1RS are hit with 95% probability

Equations given by Lander and Waterman [49] and concepts of binomial distribution in elementary probability theory were used to provide an approximation on the probability of hitting a gene by the present analysis. Assuming a size of 442×10^6^ bp of rye 1RS genome, 895,199 sequence reads with an average length of 219 bp (L), and a necessary overlap (T) of 50 bp, the probability of missing a contiguous area of 1514 bp (reported average estimated coding region of a gene; http://rice.plantbiology.msu.edu) would be approximately 4.5%, while using the elementary probability theory revealed 5.0%, respectively. In other words, the probability of hitting any 1.51 kbp region of the chromosome with at least one sequence read is 95% and with this probability nearly all genes are represented in our dataset, supposing an even distribution of the sequence reads along the 1RS genome. For obtaining an estimate on the distribution of the sequence reads along 1RS, two genes the 45S ribosomal locus and the ω-secalin gene, were analysed by the MOSAIK software suit, which provides a base-accurate coverage plot analysing the representation of every base of the reference sequence in the sequence reads. Locally assembled rye specific 45S ribosomal gene unit (JF489233) and the AF000227 sequence entry representing the ω-secalin gene repeat unit were selected as reference sequences. These genes are repeated several times in the 1RS genome therefore a sufficient number of sequence reads were available for the analysis (Table S1.). Both reference sequences cover about 9 kbp providing sufficient template for the coverage analysis. In each template there were self complementary repeat regions as revealed by dotplot analysis, yielding higher multiplicity in these regions (Supplemental Figure S1.). The ω-secalin locus additionally contained a repeat region, corresponding to the TREP 255 (retrotransposon, LTR, unknown) entry yielding elevated coverage of this region. Of the total length of the ω-secalin gene 98.4% was represented by sequence reads except for a 152 bp region. The mean coverage was about 9 fold (Standard deviation +/−4) per basepair disregarding the repeat regions, which in magnitude coincides well with the published copy number of 15 for this gene [48]. On the other part, full length of the 45S ribosomal gene was represented by sequence reads yielding about 360× (+/−71) mean coverage, not considering the intergene spacer region. The estimated copy number of this locus in the rye was about 2,000 [22] but Gustafson et al. [50] reported reduction of the NOR region for 1RS present in the wheat.

#### About three quarter of the 1RS genome is of repeated nature

Identification of repetitive elements in 1RS sequence reads was done, as described in the Material and Methods section, in several consecutive steps within the analysis pipeline. The approach comprised the identification of known repeat types, like microsatellite regions and transposon type repeats but de novo repeat elements were also identified.

#### Simple Sequence Repeats of 1RS

Non-transposon-type repeats such as SSRs were identified in 18.5% of all sequence reads (165,629 reads), amounting to 204,286 SSR sequences, which were present either as single or as compound SSRs in various combinations. However, only 2,048 of these reads (0.23%) harboured SSRs spanning more than 50% of the total length of the sequence read. These were identified as SSR reads and were not analysed further, while the rest were labelled as SSR containing reads. (AG)_n_ was the most abundant dinucleotide repeat (4.8%), followed by (AT)_n_ (3.5%). (AAG)_n_ was most abundant (0.7%) among trinucleotide repeats, and (AAAT)_n_ among tetra-nucleotides (0.2%) (Table S4_All SSRs). The frequency especially in case of tri- and tetra- nucleotide repeats may be underestimates since uncertainty at homopolymer identification of 454 sequencing can disturb the detection of true SSR regions by interrupting the regular repeat sequence.

#### SSR containing elements as genetic markers

Two categories of SSR containing reads were analysed for genetic marker identification. SSR reads (2,048 reads) and sequence reads identified as gene related but also harbouring SSRs. Nearly one fourth of the gene related reads corresponded to this latter category (7,162 reads). For establishing PCR amplicons for the SSRs the position of the SSR within the sequence read was identified. Considering an average PCR primer length of 18–22 bp we computed the proper SSR positions for minimum of 30 bp and 40 bp flanking lengths. SSR containing reads fulfilling these requirements were labelled as putative candidates for genetic markers (Table S4).

Concerning the SSR reads, 321 or 455 (15.7% and 22.2%) reads were identified dependent on the 30 bp or 40 bp permitted length of the flanking sequence (Table S4_ SSRs). This was contrasted by the gene related sequence reads, where more than half of the reads harboured the SSRs in a usable position (Table S4_Gene related SSRs). Preliminary analysis of 103 SSR reads revealed 26 exclusively 1RS specific bands.

#### Six transposon classes dominate the 1RS repeat landscape

Known type repeat element discovery was based on the TREP repeat database using its categorisation also for those elements identified by use of other databases. This way, 68.5% of all reads were tagged matching at least one of the entries (Table 2). The repeat elements of 1RS hit 341 of the 1,717 entries present in the full TREP-repeat database, including 122 Class I, 183 Class II and 36 non-classified types (Table S5). However, 4 entries of the 35 of rye origin in the TREP database yielded no homology to any of the 1RS-specific sequence reads. The identified reads included the frequently used rye-specific probes representing interspersed repeat element pSC119.1 (0.6%) and pSC119.2 (0.05%), predominantly a telomere marking probe. Also ‘Revolver’, the recently described [51] in the rye genome evenly distributed transposon represented 0.3% of the sequence reads, while Bilby (retrotransposon/LTR/Copia), the rye genome-specific centromeric repeat family [52], was represented by 0.2% of all reads (Table S5). As far as the transposon elements were concerned, the superfamilies Gypsy and Copia were the most abundant retrotransposons, constituting about 42.7% and 7.3% of the sequence reads, respectively, followed by the DNA transposon superfamily CACTA (6.3% of all reads) (Table 2).

**Table 2:** Representation of diverse repeat elements in1RS.

Repeat category			% of all reads
**Simple Sequence Repeats** *			**0.2288**
**Known repeat elements**			**68.4590**
	DNA_transposon/Helitron	DNA_transposon/Helitron/helitron	0.0139
	DNA_transposon/TIR	DNA_transposon/TIR/CACTA	6.3228
		DNA_transposon/TIR/Harbinger	0.1048
		DNA_transposon/TIR/HAT	0.0094
		DNA_transposon/TIR/Mariner	0.1916
		DNA_transposon/TIR/Mutator	0.1987
		DNA_transposon/TIR/unknown	0.0047
	DNA_transposon/unknown	DNA_transposon/unknown/unknown	0.0506
	Retrotransposon/LTR	Retrotransposon/LTR/Copia	7.2632
		Retrotransposon/LTR/Gypsy	42.6917
		Retrotransposon/LTR/unknown	0.9523
	Retrotransposon/LINE	Retrotransposon/LINE/unknown	0.3162
	Retrotransposon/SINE	Retrotransposon/SINE/unknown	0.0035
	Unknown	unknown/unknown/unknown	3.2932
	Rye revolver	revolver	0.3007
	Putative transposon elements		6.7416
**Novel repeats**			**3.686**
**All repeat sequences**			**72.3742**

*Sequence reads with more than 50% SSR sequence = SSR reads.

Half of the sequence reads identified as repeat elements were derived from 29 transposon families, of which 25 represented Class I retrotransposons (Gypsy 21 and Copia 4) and 4 families Class II DNA transposon types (all CACTA). The most abundant family in 1RS was the Class I retroelement Gypsy/Sabrina, comprising 13.3% of the sequence reads and outnumbering about 2.5 fold the second most abundant one, namely Gypsy/WHAM (4.9%). Further abundant retroelements were Wilma, Sabine and Cereba (all of the Gypsy superfamily) amounting to 3.3, 2.8 and 2.5% of the sequence reads, respectively. The CACTA superfamily Jorge was the most frequent Class II DNA transposon element; accounting for 2.7% of the sequence reads (Table S5).

Sequence reads partially fulfilling the filtering criteria (good alignment, but low coverage, or poor alignment, but good coverage) but not tagged otherwise were also considered as putative repeat elements representing 6.7% of all reads (Table 2).

#### Novel repeat elements

RepeatScout a de novo repeat finding software suite [53], was used for the identification of putative repeat elements. This system identified 9,842 putative repeat sequence elements after elimination of low complexity and tandem repeats. Realigning the derived putative repeat motifs with the sequence reads using RepeatMasker software yielded 685 repeat sequences, hit by at least 10 sequence reads. These were accepted as “novel repeats”. Finally, to eliminate multiple representations among the novel repeats BASTClust was used reducing the number of identified novel repeat to 638 represented by 31,001 sequence reads. The novel repeats had an average length of 245 basepair (ranging 51–461 bp) and were represented by 10 to 317 sequence reads.

#### Chimeric sequence reads indicate putative chloroplast insertions in 1RS

Analysis of the nuclear genomes of plants revealed insertions of genes originating from organelles into the nuclear genome [54], [55]. For assessing the presence of organelle-specific sequence reads indicative of putative insertions in 1RS, the dataset was screened firstly with high stringency parameters (90% overlap and 90% identity) against the available chloroplast and mitochondrial sequence of wheat, identifying 0.2% of all reads (1803 hits). Within the hit sequences, 421 represented chloroplast (Cp) and 1355 mitochondrial (Mt) sequences, while 27 could not be exclusively assigned to either of the genomes (Table S1). Secondly, screening with equally high identity value (90%) but with reduced overlap (40%) was done to identify putative chimeric fragments representing organellar/nuclear junctions harbouring both organellar and putative 1RS specific DNA sequences. This latter screening revealed no mitochondrial, but 24 sequence reads representing 15 chloroplast regions of chimeric type. Six out of the 15 regions represented chloroplast/transposon junctions. One represented a junction annotated as Triticum aestivum chromosomal DNA, while 8 of them were junctions of chloroplast to no-homology sequence regions. Controlling the validity of these junction regions by PCR, 9 out of the 15 reamplified from a mixture of different sorts of rye genomic DNA (3, 1 and 5 positive PCR reactions in junctions of chloroplast/transposon, chloroplast/chromosomal and chloroplast/no-homology,respectively). The existence of chimeric fragments indicates the presence of chloroplast genome insertions into 1RS, while similar mitochondrial ones could not be verified. The organelle specific sequence reads covered 54% of the chloroplast and 49% of the mitochondrial genomes, yielding somewhat higher values than the average obtained for the whole dataset (43%). The presence of contaminating organellar DNA, however, can not be excluded completely especially in case of mitochondria.

#### Miscellaneous non-coding genomic regions

For recovering all possible matches of the sequence reads to the available plant sequence information the sequence reads were tested against databases representing miscellaneous sequence information of non-coding genomic regions, such as *Brachypodium distachyon* and rice databases representing 1 kbp upstream, downstream regions of genes or intergenic regions. In all 79,271 sequence reads were identified, representing nearly 8.9% of all reads of functionally non-identified regions. The majority of these (8.47%) hit 374 BAC NCBI entries (37 from *Hordeum*, 337 from *Triticum*) while the rest were characterised by the *Brachypodium* and rice databases representing 1 kbp upstream, downstream and intergenic regions of the genes and non-BAC NCBI entries (SCAR, microsatellite, RAPD, STS marker) (Table S1)

### Relation of 1RS to other grass genomes

#### Synteny to *Oryza sativa* and *Brachypodium distachyon* reveals uneven distribution of loci

**Figure 2:**
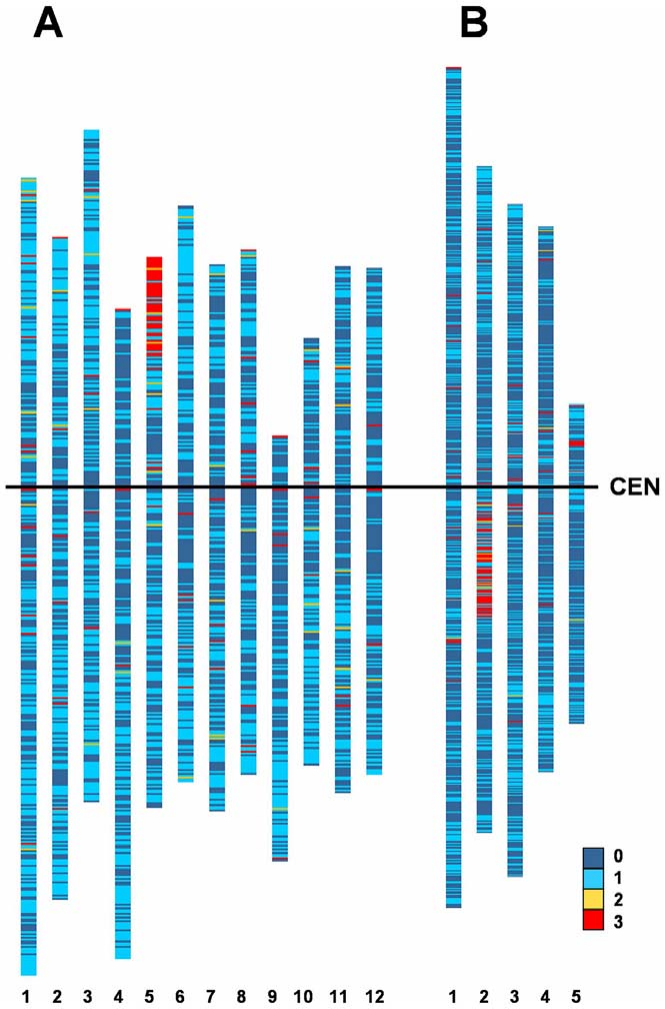
Synteny of 1RS sequence reads with the non-TE related gene models of *Oryza sativa* and *Brachypodium distachyon*. The frequency of gene hits, in the in silico generated bins of the *Oryza sativa* and *Brachypodium distachyon* genomes, were expressed as multiples of standard deviation (SD). **A**: *Oryza sativa* chromosomes 1–12. **B**: *Brachypodium distachyon* chromosomes 1–5. **CEN**: Centromere. **Colour coding:** **0** Bin with no 1RS homology. **1** Bins with non-significant 1RS homology **2** Bins with significant 1RS homology at 95% level (1.98 SD) **3** Bins with significant 1RS homology at 99% level (Highly Homolog Bin, 2.58 SD).

To visualise the synteny (gene content identity) of the 1RS genome to the genomes of rice and *Brachypodium distachyon,* the model genomes were dissected in silico into 10^5^ bp bins, resulting in 3,729 and 2,713 bins, respectively. On average, these bins harboured 10.9 and 9.4 gene models per bin for *Oryza sativa* and *Brachypodium distachyon*, respectively (Table 3). Subsequently, rye sequence reads previously identified as putative genes in rice and *Brachypodium distachyon* genomes were allocated to these bins, based on the common rice annotation for the loci referring to the rice LOC_Os loci (Rice Genome Annotation database). In this way, 3,076 gene models of rice and 1,363 of *Brachypodium distachyon* were recognised in the two model genomes as homolog to rye 1RS sequence reads. Homolog genes to 1RS were dispersed throughout the genomes, including 1,863 (49.96%) and 931 (34.3%) bins in *Oryza sativa* and *Brachypodium distachyon* genomes, respectively (Fig. 2). Identifying the bins harbouring high proportion of homolog loci to 1RS (Highly Homolog Bin, HHB) allowed us to identify regions of the rice genome preferentially represented in 1RS. The Highly Homolog Bins possibly represent group of genes, which were inherited as blocks during evolution. We identified 109 HHBs in *Oryza sativa* and 100 HHBs in *Brachypodium distachyon*, containing 436 and 306 gene model hits, respectively (Table 3). In both *Oryza sativa* and *Brachypodium distachyon*, almost every second gene model hit fell into ‘highly homolog bins’: 43.56% and 44.41% of the genes, respectively.

**Table 3:** Synteny of the 1RS chromosome arm to rice and *Brachypodium distachyon* genomes.

	Rice	Fraction in %	Brachypodium	Fraction in %
**Total number of bins**	3729	100.00	2713	100.00
**Average number of gene models/bin**	10.88		9.41	
**Bins hit by sequence reads**	1863	49.96	931	34.32
**Number of preferential bins**	109	2.92	100	3.69
**Number of gene models in the genome**	40588	100.00	25534	100
**Number of gene models hit by sequence reads**	3076	7.58	1363	5.34
**Total number of gene models/preferential bins**	1001	100.00	689	100.00
**Number of gene models hit in preferential bins**	436	43.56	306	44.41

Bins showing homology to sequence reads were distributed on all chromosomes of both model genomes (Fig. 2) while HHBs showed clustering in both genomes. In rice about 42% of HHBs concentrated in the distal region of the short arm of chromosome 5, while in *Brachypodium distachyon* more than one half (54%) of the HHBs clustered in the proximal region of the long arm of chromosome 2 (Table 4). In these HHBs there are 546 rice and 502 *Brachypodium distachyon* gene models, while only 262 (48%) and 232 (46.2%) were hit by the 1RS related sequence reads.

**Table 4:** Distribution of Highly Homolog Bins (HHB) on the chromosome arms of rice and *Brachypodium distachyon.*

Chromosome arm	Number of bins	Number of HHB	% of all HHB
***Rice***			
chr01S	169	6	5.6
chr01L	264	7	6.5
chr02S	137	2	1.9
chr02L	223	5	4.6
chr03S	195	3	2.8
chr03L	170	2	1.9
chr04S	98	1	0.9
chr04L	255	2	1.9
**chr05S**	**126**	**45**	**41.7**
chr05L	173	1	0.9
chr06S	154	0	0.0
chr06L	159	5	4.6
chr07S	122	0	0.0
chr07L	175	4	3.7
chr08S	130	6	5.6
chr08L	155	3	2.8
chr09S	29	1	0.9
chr09L	202	4	3.7
chr10S	82	3	2.8
chr10L	150	2	1.9
chr11S	121	1	0.9
chr11L	165	2	1.9
chr12S	120	2	1.9
chr12L	155	2	0.9
***Brachypodium***			
chr01S	375	7	7.0
chr01L	374	7	7.0
chr02S	287	6	6.0
**chr02L**	**307**	**54**	**54.0**
chr03S	253	7	7.0
chr03L	346	5	5.0
chr04S	233	5	5.0
chr04L	253	3	3.0
chr05S	75	6	6.0
chr05L	210	0	0.0

#### Synteny analysis of 1RS to 1HS identifies conserved blocks of genes

**Figure 3:**
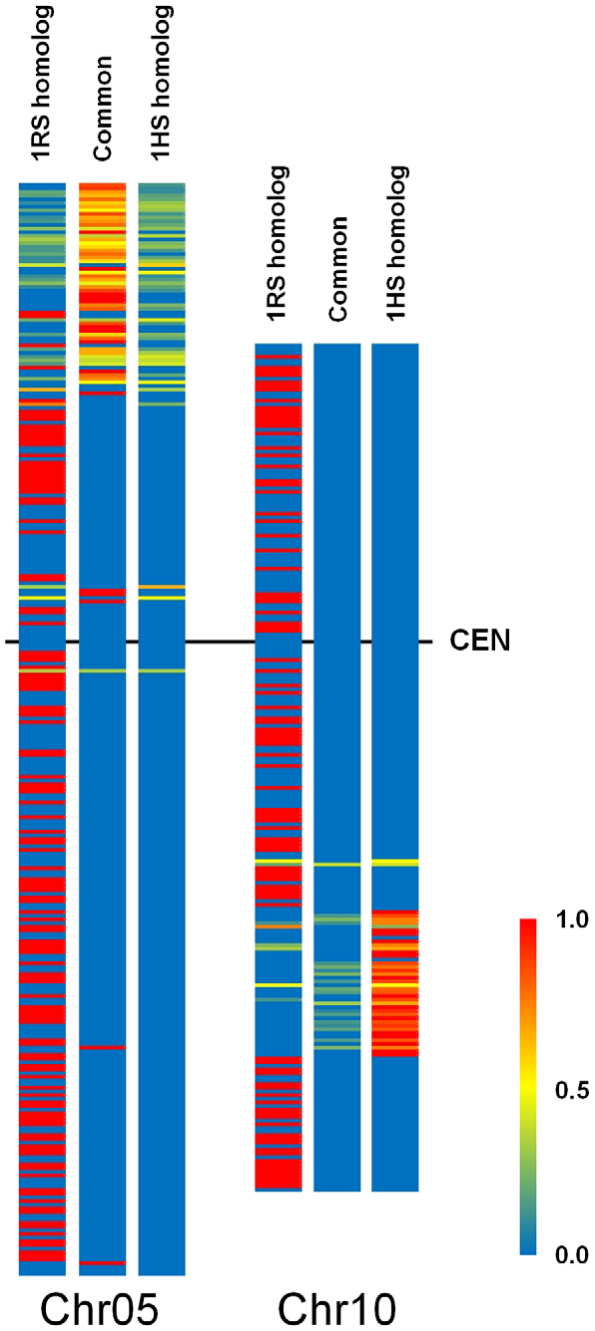
Comparison of the gene content of 1RS and 1HS based on Chr.5 and Chr.10 of the model rice genome. The in silico generated 10^5^ bp virtual bins of chromosomes 5 and 10 of rice were used as platforms to compare the gene content of 1RS and 1HS homolog to rice. Similarity of 1RS to 1HS concerning a particular rice bin was judged by estimating the portion of common loci over all homolog loci in a particular bin as described in Material and Methods. The colour bar shows the level of similarity starting from 0 (blue) to 1 (complete similarity; red) meaning that all hit loci show homology to both 1RS and 1HS. **Common**: distribution of bins containing genes homolog to both 1RS and 1HS. **1RS homolog**: bins containing genes homolog to 1RS, **1HS homolog**: distribution of bins containing genes homolog to 1HS. In the two latter cases red colour defines bins with genes representing exclusively either 1RS or 1HS homologs.

Sparse availability of sequence based mapped markers of 1RS in comparison to 1HS did not allow a direct collinearity (gene order) analysis of the two chromosomes. However, the gene content of 1RS was correlated to that of 1HS based on rice as reference genome. Recently, collinearity analyses of chromosome 1H of barley to rice and *Sorghum* genomes was published [41] describing 4,125 rice loci homolog to genes identified on 1H. More than one third of these genes (1,409) were located on rice chromosomes 5 and 10. In a latter publication, this number was revised to 1,845 [42]. Using this dataset, we identified 322 and 218 homolog loci on the short arm of chromosome 1H, homolog to the genes identified on rice chromosomes 5 and 10, respectively. In addition, we identified 465 rice 1RS homolog loci on chromosome 5 and 156 on chromosome 10 (Table S6.). The homolog rice gene sets corresponding to 1RS or 1HS were compared analysing small increments of the rice genome by using the 10^5^ bp virtual bins described above, which allowed the identification of gene sets with high similarity. The similarity index of a bin was calculated by comparing the number of genes showing homology to both 1HS and 1RS (common genes) to all genes showing 1HS and/or 1RS homology in that particular bin (see M&M). Regarding chromosome 5 related gene set, 242 common genes (44.4%) out of the 545 homolog loci were identified. These were dispersed in 57 bins (average similarity 0.77). Concerning chromosome 10 only 24 (6.9%) common genes located in 19 bins (average similarity 0.18) were identified out of the 350 homologs (Table 5). As far as the distribution of the homolog genes on the rice chromosomes were concerned, chromosome 5 specific common homologs cumulated in bins on the distal part of the short arm. Bins containing common genes related to chromosome 10, clustered in the middle of the long arm (Fig. 3). The marked difference, both in the proportion of common genes and levels of similarity, suggests that chromosome 5 related blocks of genes are evolutionarily more conserved compared to chromosome 10 related elements, which cluster in the pericentric region of 1HS. Additionally there is marked difference between the 1HS and 1RS genomes concerning the distribution of those genes, which are not common in the two chromosome arms. Exclusively 1RS specific rice homologs are distributed along the full length of rice chromosomes 5 and 10. Only occasional representations of exclusively 1HS specific rice homologs were recorded on chromosome 5. On chromosome 10 1HS specific rice homologs were confined to the region of common genes (Fig. 3).

**Table 5:** Comparison of gene content of 1RS and 1HS based on homology to the rice chromosomes 5 and 10.

Rice chromosome	Number of homolog loci					All homolog loci
	1RS	1HS	1RS specific	Common	1HS specific	
**Chr.05**	465	322	223	242	80	545
**Chr.10**	156	218	132	24	194	350
**All**	621	540	355	266	274	895

## Discussion

High-throughput 454 sequencing proved to be a powerful means of gaining deeper insight into the sequence composition of huge plant genomes by providing an enormous quantity of sequence information at a reasonable cost [56], [57]. Recent progress in dissecting plant genomes to small parts by chromosome sorting, using flow cytometry coupled with high-throughput DNA sequencing technology [39], [41]–[43] has provided new and powerful means of analysing complex gigabase-sized plant genomes. To date, this approach has been used to sequence the barley chromosome 1H [41] and chromosome 7DS of wheat [43]. Using a similar approach described by Mayer et al. [41], in the present study we focused on the short arm of chromosome 1R of rye, which carries a number of known agriculturally important genes and has been included in a large number of wheat varieties as 1BL.1RS translocation. Our results provide the first large-scale insight into the sequence structure and composition of the rye genome and 1RS, in particular. Moreover, we showed that next-generation sequence read coverage as low as 0.43× may provide valuable insights into the genome of interest, allowing the identification of genes present in the genome with an estimated probability of up to 95%.

In this work we used Roche 454 FLX technology to sequence DNA derived from flow-sorted chromosome 1RS. To provide sufficient amounts of DNA for sequencing the DNA of the sorted chromosomes was amplified by Multiple Displacement Amplification. Despite the fact that MDA has the lowest amplification bias among whole genome amplification methods [58] it has its limitations in quantitative analysis by uneven amplification of certain repeat elements [59]. Therefore, the quantitative interpretation of the present results especially concerning the repeat elements should be done with caution. However, comparative analysis of the major repeat classes with 454 sequence reads (kindly provided by Dr. A. Houben) representing the whole rye genome (∼0.04× coverage) without MDA yielded only minor differences, which may be accounted for either by the existing difference between the whole genome and 1RS, or the difference in the coverage (Data not shown).

In the absence of a reference genome, the biggest challenge in 454 fragment analyses is the alignment of sequence reads to entries of the diverse and largely heterologous databases by BLAST. Even with large sequence databases whenever the length of the input sequence is very short (i.e., 100 bp and below), the e-value tends to be very high (e^−2^ and above) even with perfect alignment. The reason for this is that a shorter alignment is much more likely to be coincidental than a longer one. Particularly for 454 sequences this may result in missing significant hits when using the e-value for hit evaluation, as even a high overlap (of up to 100%) or high identity (between 90 and 100%) would not be reflected adequately in the e-value. For this reason, the criteria based on overlap (at least 55%), as well as identity (at least 80%) and alignment length of at least 60 bp or 20 aa, respectively, were introduced. However, by the use of these criteria, good alignments of longer sequence reads (the longest 454 fragments had up to 350 bp) would have been missed inadvertently because of the strict identity parameter of (minimum) 80%. Therefore, the e-value approach of using a limit of e^−20^ with a lowered identity (70%) was introduced as a secondary parameter set. According to our experience, the different BLAST algorithms (BLASTn, BLASTp, BLASTx, tBLASTx) are more sensitive to implementation differences than identity/overlap-based filtering, which was applied as the primary parameter set. Therefore, it was justifiably assumed that the BLAST results would be highly comparable.

Nearly 200 million bp sequence information was obtained in the course of this work, yielding about 0.43-fold coverage of the 1RS chromosome arm. Despite the relatively low coverage, a chance of hitting a gene was estimated to be 95%. The fairly uniform coverage of the 45S rDNA and the ω-secalin genes supports this estimate and suggests that theoretically nearly all of the gene loci present in 1RS were identified. However, the identification of genes in the dataset may be hampered by the evolutionary distance of the query sequences and the used heterologous sequence databases, resulting in lower recovery of genes. To resolve this problem at least in part, we used several sequence databases of closely related species. One approach estimating the putative number of gene loci on 1RS is to sum up the number of non-overlapping gene loci hit in different databases accounting for gene space. By this procedure, we estimated the number of gene loci in 1RS to range from 3,121 to 6,759, including the secalin genes. However, considering contamination of the sorted 1RS fraction by various wheat chromosomes, we may also predict an overestimation of about 10% for the gene count. Furthermore, the annotations arising from UniGene databases must be treated with caution, because these databases harbour EST entries. Thus, more than one Unigene entry may represent the same gene locus. The entries may also be related to transposable elements and duplicates of genes can exist in the different species specific datasets. All these factors may further reduce the estimated highest number of gene loci on 1RS.

Provided 1RS comprises about 5.6% of the rye genome and harbours at least 3,121 gene loci, an estimated 56,000 gene loci per haploid rye genome may be assumed, disregarding the rDNA loci. This is in good agreement with the dimension of nearly 51,000 identified gene models in *Oryza sativa*, (http://rice.plantbiology.msu.edu/), 38,000 to 48,000 genes in barley [41], [42] and 55,000 to 111,000 gene loci per diploid genome of wheat [60]. On the other hand, we identified at least 1,882 different gene functions linked to 1RS, which is certainly an underestimation of the true number of gene functions, because 3,852 expressed and hypothetical genes were identified with no functional annotation. Among the identified gene functions, several loci involved in powdery mildew resistance were found in 1RS, which may be responsible for conveying that resistance to the wheat cultivars. In our dataset all the eleven genes recognised by Bartos et al. [21] in 1RS using BAC end sequence information were recovered.

The analysis of sequence composition of 1RS offered an opportunity to focus on the representation of organellar genome elements. In eukaryotes, DNA has been, and still is being exchanged between endosymbiosis-derived mitochondrial and chloroplast genomes and the nucleus, thus serving as a significant driving force for gene and genome evolution [61]–[63]. Organelle-to-nucleus DNA transfers during early organelle evolution generated massive relocation of organelle genes into the nucleus, resulting in functional entities still being actively transcribed. In contrast, nearly all recent nuclear transfers of mitochondrial or plastid DNA concern non-coding sequences. For the rice genome, organellar insertions have been reported to possibly occur in hotspots where whole blocks of organellar DNA insertions can be found [64]. The present study revealed the presence of chloroplast insertions in 1RS by identifying chimeric sequence reads of chloroplast and putative genomic elements. Despite the higher number of identified mitochondrial specific sequence reads, chimeric fragments with mitochondrial sequences were not detected, suggesting possible mitochondrial contamination of the sorted chromosomes.

Our results indicate that 74.46% of the 1RS chromosome arm sequence comprises known repeat elements, including the ribosomal loci, but omitting putative gene families. These data concur well with those reported by Bartoš et al. [21], based on analysis of BAC end sequences (BES) of clones representing 1RS (75.6% known repeats). However, there were differences in the subclasses e.g. Gypsy 49% vs. 43%, Copia 14% vs. 7.3% and CACTA 4.4% vs. 6.3% comparing BES and the present dataset. This difference may be attributed to the about 100-fold difference in the coverage: 0.45% versus 43% coverage of the 1RS genome for the BES and the present dataset, respectively. Additionally, Bartoš et al. [21] found 8.8% unknown repeat elements, while in our case we identified about 4% novel repeat elements by RepeatScout search resulting in 84.2% and 74.5% of repeat elements, respectively. However, the data based on 1RS sequence information are markedly below the 92% rate published earlier by Flavell et al. [20]. In the latter work, the portion of repeated sequences of the whole rye genome was determined by C_o_t analysis, which identifies all repeat elements within a genome, while BES and our strategy identified repeat elements using in silico techniques only. The deviation of in silico obtained data from those previously reported by Flavell et al. [20] may be due to the difference in methodology, C_o_t analysis is more sensitive identifying repeat sequence elements in a genome.

The obtained sequence information may also be exploited for generating genetic markers for this chromosome arm. This way we identified about 5,000 putative marker sites based on SSR regions either spanning more than half of the sequence read or present in a sequence read representing gene space of 1RS. Preliminary experiments showed that about one fourth of the identified regions may serve as 1RS specific markers on PCR basis in 1BL.1RS translocation wheats. These amplicons are possibly applicable also in diploid rye. Additional to SSR based marker sites all sequence information related to genes in 1RS may be exploited to generate for example SNP or InDel type markers by resequencing.

The colinearity of plant genomes was discovered after the advent of comparative mapping, culminating in the establishment of comparative genomics. The most comprehensive dataset in the plant kingdom is available for the *Poaceae* family, including the staple cereals maize, wheat, barley, rye, sorghum and millets, along with rice and *Brachypodium distachyon*. The last two species are fully sequenced small genomes [65], [66]. In our study, the annotated 454 sequence reads were not genetically mapped on the rye chromosome arm. However, the large number of putative gene loci permitted a detailed genome-wide correlation analysis of 1RS to the full genomes of rice and *Brachypodium distachyon*. The analysis revealed that genes present in 1RS are scattered through the entire length of the two model genomes, but not evenly. In accordance with previous observations on the colinearity of rice chromosome 5 and rye chromosome 1 [37], [38], in our case highly homolog bins representing syntenic regions clustered in the distal region of the short arm of rice chromosome 5 and in the proximal region of the long arm of chr2 of the *Brachypodium distachyon*. This concurs with the high colinearity between rice and *Brachypodium distachyon* in these two regions. Additionally in the present study we show the first synteny analysis within Triticeae between rye and barley based on data generated by next generation sequencing technology. The comparison of the gene content of the short arms of chromosome 1 was made via the sequence information available for the rice genome used as platform since full sequence information of the compared genomes is not available. Using virtual bins generated on the model genome revealed genome sections which are inherited in blocks during evolution of these species. The synteny analysis of the short arms of chromosome 1 of the two species revealed that more compact regions of the rice genome were represented in 1HS, while homolog regions of 1RS were more scattered in the analysed rice chromosomes. The highest similarity of the 1RS/1HS gene content was localised on the distal part of rice chromosome 5 proposing high level of conservation in this region. Much lower similarity was observed regarding chromosome 10 related genes, which represent the pericentric region of 1HS. The observed difference between the two chromosome arms may be related either to the approximate position of the centromere in 1H or to the lower conservation of this region during evolution. We have to note however, that the different approach in homology identification in 1RS and 1HS may also contribute to the observed differences.

### Conclusion

Due to its presence in many varieties of wheat grown worldwide, the rye chromosome arm 1RS is considered an important element of the wheat germplasm. The present study revealed the gene content and potential gene functions on this chromosome arm and demonstrated numerous sequence elements such as SSRs and gene-related sequences which can be utilised for future research as well as in breeding both wheat and rye. Recently, BAC-fingerprinted contigs of 1RS were also established (Burg K, unpublished) and deposited in a database for physical maps hosted by UC Davis (http://probes.pw.usda.gov:8080/rye1RS/). Tsuchida et al. [67] developed 1RS deletion (dissection) lines which have been successfully used to physically map 150 1RS-specific SSR markers 26 originating from the present study into 15 bins (Lelley T, personal communication). In the present study we correlated the gene content of 1RS to 1HS *Hordeum* chromosome arm, which is genetically a well studied component of the Triticeae genomes. All of these results and resources will contribute to further describe the molecular structure of 1RS. As all chromosomes of rye can be purified by flow cytometric sorting, either directly from rye (chromosome 1R) or from wheat-rye chromosome addition lines - chromosomes 2R–7R [44], the strategy outlined and verified in the present study may be used for sequencing and detailed analysis of the entire rye genome in a stepwise manner for each chromosome.

## Materials and Methods

### Chromosome sorting and multiple displacement amplification (MDA)

Seeds of a 1RS wheat-rye ditelosomic addition line, derived from rye (*Secale cereale* L.) cv. ‘Imperial’ in the background of wheat (*Triticum aestivum* L.) cv. ‘Chinese Spring’ [68], were provided by Dr. B. Friebe (Kansas State University, Manhattan, USA). As this 1RS addition line is cytologically unstable, seeds selected for propagation were checked for the presence of 1RS by cytology in root tips. Only those seeds possessing 44 chromosomes, including the two telocentrics of 1RS were grown. Plants from such seeds produce about 60% ditelocentric and 40% monotelocentric progenies, while monotelocentric plants with 43 chromosomes (42+1 telocentric 1RS) would only produce about 30% monotelocentric and no ditelocentric progenies, rendering them unsuitable for sorting. Seeds were germinated up to an optimal root length of 2–3 cm. Cell cycle synchronisation and accumulation of metaphases in root tips and preparation of liquid chromosome suspensions were performed according to Kubaláková et al. [44]. Chromosomes in suspension were stained by 2 µg/ml DAPI (4′,6-diamidino-2-phenylindole) and analysed using a FACSVantage SE flow cytometer (Becton Dickinson, San José, USA) at a rate of about 800–1200 chromosomes/sec. Telosome 1RS was sorted at a rate of 10/sec in batches of 30,000 into 50 µl deionised water in a PCR tube. The purity in sorted fractions was checked by fluorescence *in situ* hybridisation with chromosomes sorted onto a microscope slide using probes for subtelomeric heterochromatin (pSc200) and telomeric repeats [40]. Isothermal amplification of chromosomal DNA was performed according to Šimková et al. [40]: flow-sorted chromosomes were treated with proteinase K, purified using a Microcon YM-100 column (Millipore Corporate, Billerica, USA), and the purified chromosomal DNA was amplified using the illustra GenomiPhi V2 DNA Amplification Kit (GE Healthcare, Chalfont St. Giles, United Kingdom), according to manufacturer's instructions, in a 20 µl reaction for 1.5 hours. The samples were lyophilised and shipped for 454 sequencing.

### Roche 454 FLX runs

Five micrograms of DNA amplified from flow-sorted 1RS were used to prepare a 454 sequencing library using the GS FLX DNA library preparation kit in accordance with the manufacturer's instructions (Roche Diagnostics, Branford, USA). Single-stranded 454 sequencing libraries were quantified by a quantitative PCR assay [69] and processed using a GSFLX standard emPCR kit I and a standard LR70 sequencing kit (Roche Diagnostics, Branford, USA) according to manufacturer's instructions. Sequencing was performed on four lanes of a 16-lane gasket on a 70×75 FLX picotiter plate (“titration”) and on two complete 70×75 picotiter plates, resulting in 942,768 reads with a mean read length of 219 bp, yielding ∼205 Mb of raw sequence data. Data of the two full runs along with the titration run were pooled in order to process the entire dataset in a single batch.

#### Sequence Analysis

Low-quality sequence reads were removed by the Roche 454 integrated NEWBLER (v1.1.03.24) software. A bioinformatic pipeline was established to analyse the remaining sequence reads. Various software tools and Perl scripts were integrated for this purpose. The core of the result documentation was a Microsoft SQL Server database, which was used to collect all analysis outputs and store analysis data for subsequent result queries.

The pipeline utilised a stepwise procedure to classify sequence reads, starting with the elimination of fragments shorter than 50 bps. The remaining sequence reads were scanned for perfect and nearly perfect duplicate sequences using PHRAP [70] to remove technical artefacts. Two sequence reads were considered as perfect duplicates when they were of identical length and no single base mismatch was found in the alignment of the two sequences. Sequence reads were considered nearly perfect duplicates if they showed (1) less than 5 bp length difference, (2) maximum of three mismatches and (3) maximum of 2 bp offset in alignment. Sequence reads fulfilling either one of the two matching criteria were considered as technical duplicates and were removed, leaving only one representative of the sequence reads in the dataset. After removing the technically inappropriate reads, the sequence quality of the remaining sequence reads was checked by analysing ‘PHRED-compatible quality values’ [71] derived from the flowgrams. These checks revealed that 99.1% of the sequence reads had scores above 20, and 0.86% between15 and 20. As few as 169 sequence reads had scores of 10–15 and only 5 were below 10.

After removal of low-quality sequences, the remaining sequence reads were considered as a representative dataset for 1RS, which was subsequently analysed for the presence of repeat, organellar or gene space elements of the genome as described below. After each step, the newly classified sequence reads were labelled with their respective classification and removed from the dataset, i.e. excluded from the next analysis steps.

### SSR identification

The representative dataset was subjected to a two-step SSR analysis using the SciRoKo software [72]. First, mononucleotides longer than 8 bp and dinucleotides with >4 repetitions of the motif were identified. Secondly, trinucleotides with >4 repetitions and tetra-, penta-, and hexanucleotides with >3 repetitions of the motif were identified. Only fragments with SSR regions spanning more than 50% of the fragment length were excluded from further analysis; the remaining sequences were tagged as SSR-containing elements and preserved in the dataset for downstream analysis.

### Repeat Element and rDNA Detection

Repeat elements and rDNA were identified using the RepeatMasker software [73], with standard parameter settings except that the checks for bacterial insertion were disabled. Three datasets were used as reference databases for repeat element detection: a) TREP, b) TIGR *Oryza* repeats, and c) a self-compiled collection of *Secale* Revolver elements. Three datasets were selected for rDNA detection: a) all rDNA elements of MIPS-REcat, b) all rDNA elements of the TIGR *Oryza* repeats, and c) a self-compiled collection of *Musa* and *Secale* rDNA elements (Table S2). Automated classification of alignments between rye sequence reads and reference sequences was based on the following criteria: alignment was deemed unacceptable if a) perc_div (percentage divergence) was higher than 30% or b) perc_div was between 20 and 30% and either perc_ins (percentage of insertions) or perc_del (percentage of deletions) were higher than 5%. In addition, alignments with less than 20 bp overlap were deemed unreliable. All other alignments were accepted as quality hits. All sequence reads covered by more than 60% quality hits were classified as repeat elements or rDNA and excluded from downstream analysis. All sequence reads fulfilling the quality criteria but either having less than 60% coverage or poor alignment were tagged as putative elements in their respective category and were kept in the dataset. At the end of the analysis pipeline, tagged sequence reads with no allocated function were assigned to the putative repeat category. Subsequently the unclassified sequence reads were further analysed by BLAST against all *Secale*, *Triticum*, and *Hordeum* sequences of the NCBI NT Cereal database (Table S2), which contains significant quantities of transposon-related sequence information. Sequence reads producing a significant hit (details on the applied parameters are given below) against transposon or rDNA elements were identified and also removed from downstream analysis.

Transposon related expressed sequences were identified by use of the Rice Protein database searching the FASTA initial line for “transposon” entry.

All repeat elements identified by the latter databases were fitted into the nomenclature system of the TREP database.

### Organellar elements

Organelle-related sequence reads were identified by BLAST comparison against the available chloroplast and mitochondria sequences of *T. aestivum* (Table S2). WU-BLAST was employed for sequence comparison, using 90% as the cut-off value for both, overlap and identity. Sequence reads producing a hit with one of the two reference sequences were classified as organellar elements and subsequently removed from the dataset. Second round of the search was made by lowered overlap value (40%) in order to identify putative chimeric sequence reads harbouring organellar as well as 1RS related genomic elements.

### Unified (annotation) ontology based on rice

To obtain a common annotation for all sequence reads consistent between species, all reference sequences available in the *Brachypodium distachyon* peptide database, *Secale cereale* UniGene, *Triticum aestivum* UniGene, *Oryza sativa* UniGene and *Hordeum vulgare* UniGene database entries were annotated against the *Oryza* peptides database (i.e. Rice Genome Annotation, RGA) using either BLASTp or BLASTx with the parameters described below. Additional annotation sources were used for loci which could not be annotated on the basis of the RGA datasets. Some of the entries of the *Triticum* UniGene and *Hordeum* UniGene databases already had an annotation by NCBI, which were accepted as additional annotation sources for the rye sequence reads. Subsequently, the remaining un-annotated entries were BLAST searched against the entries of RAP-DB (Rice Annotation Project-Database), yielding further annotated database entries. Using this approach, approximately 10% of the UniGene loci could be annotated via RGA, 2% via NCBI and 1% via RAP-DB.

### Discovery of sequence reads representing gene space

The still unclassified sequence reads of the dataset were subjected to a series of WU-BLAST 2.0 analysis. Eleven datasets were used to identify sequence reads representing the gene space of 1RS (Table S2). For gaining information on the gene space of 1RS, we first identified the well known gene loci of secalins (ω-secalin and γ-secalin). Then eight reference datasets were used to identify sequence reads representing putative expressed parts of the genome (*Oryza sativa* peptides, *Brachypodium distachyon* peptides, NCBI: *Oryza sativa* UniGene, *Secale cereale* UniGene, *Triticum aestivum* UniGene, *Hordeum vulgare* UniGene, NT Cereal and bin-mapped loci of short arms of wheat chromosomes 1A, 1B and 1D). An additional two datasets were used to elucidate putative non-transcribed regions of 1RS gene space (*Oryza* UTR, *Oryza* Intron). Only the *Secale* UniGene dataset was species specific, and was therefore aligned to the available sequence reads using BLASTn. Comparison of sequence reads to other species was performed primarily at the protein level using BLASTx on *Oryza sativa* and *Brachypodium distachyon* Peptide databases. To optimise the recovery of sequence read annotations, both tBLASTx and BLASTn were used to identify gene space specific sequences in *Oryza*, *Triticum*, *Hordeum* UniGene and NT Cereal databases, where both subject and query sequences were at the nucleotide level. The overlaps between the two methods were found to be reasonably high. Contrary to our expectations, by using tBLASTx only 10% of additional high-quality hits were identified (data not shown).

For the automated analysis of the BLAST results, it was essential to use the same set of parameters for all sequence comparisons. Alignment of two sequences was considered to be significant when i) either they had an overlap of at least 55%, identity of at least 80% and an alignment length of at least 60 bp or 20 aa respectively, or alternatively ii) they had an e-value of e^−20^ and identity of at least 70%. The first criterion was used for primary filtration of the dataset followed by criterion 2 with subsequent pooling of the hits. The first hit fulfilling these criteria was used as annotation for a given sequence read, revealing a single annotation per read.

### Synteny analysis of rye 1RS to Oryza sativa and Brachypodium distachyon genomes

To visualise the synteny of the 1RS sequence to the genomes of rice and *Brachypodium distachyon,* the model genomes were dissected in silico into 10^5^ bp bins. Subsequently, sequence reads previously identified as putative genes in the corresponding genome were allocated to the generated bins. For each bin the raw score (X = number of loci hit by at least one sequence read divided by the total number of loci in the bin) was identified. To compare the bins, the standard score (Z) for each bin was determined according to the following formula:
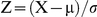
(X = raw score, μ = mean value, σ = standard deviation). To identify bins and regions of the model genomes being overrepresented in 1RS, *highly homolog bins* (HHBs) with standard scores over 2.58× standard deviation (σ) (99% limit) were selected, and their distribution in the model genomes was analysed.

### Comparative gene content analysis of 1HS and 1RS

For comparison of the gene content of 1RS and 1HS the fraction of the common homolog gene content (Similarity) of the 10^5^ bp virtual bins was calculated according to the following formula:
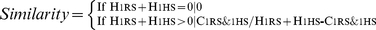
C_1RS&1HS_: number of rice loci showing homology to both 1RS and 1HS specific sequence reads (Common loci). H_1RS_: number of rice loci homolog to 1RS specific sequence reads. H_1HS_: number of rice loci homolog to 1HS specific sequence reads.

Calculating the fraction of the homolog but not common loci related to either 1RS or 1HS the similar formula was used except the numerator was changed to either H_1RS_-C_1RS&1HS_ for 1RS or H_1HS_- C_1RS&1HS_ for 1HS.

Conversion of the RAP-DB loci identifications published in Mayer et al. [42] to the corresponding RGA loci was done using the RGA-MSU conversion table (http://rapdb.dna.affrc.go.jp/download/index.html). The putative centromere position in 1H was identified according to the UCR04162008 map set of the *The Hordeum Toolbox* (http://wheat.pw.usda.gov/tht/maps.php). Based on these, 300 chromosome 5 and 168 chromosome 10 homolog RGA loci were identified representing the 1HS genome (Table S6).

### Probability estimation of hitting single genes in 1RS

In order to estimate the probability of hitting a single gene both the Lander and Waterman equations [49] and the elementary probability theory were applied. Using the Lander and Waterman formulas, the probability that a gene of an average length (L_g_ in bp) is not covered by any sequence reads contained within the current dataset was calculated by the following assumption: the probability for an “ocean” (a gap between two detected sequences) of the length kL is given as *e^−c(k+θ)^*, where L is the length of an average sequence read, k is a sizing factor (L_g_/L), c = (N * L)/G the redundancy of coverage, and θ = T/L. (N = number of sequence reads, L = average length of sequence read in bp, G = length of the investigated genome in bp and T = the desired length to detect overlap in bp). Using the elementary probability theory and applying concepts of binomial distribution, the probability of a given portion of the genome being not hit is given as (1−L/G)^N^, which is approximately equivalent to e^−c^ for large N.

### Coverage plot of ω-secalin and 45S rDNA loci

MOSAIK - http://bioinformatics.bc.edu/marthlab/Mosaik - a reference-guided assembler, was used to map all sequence reads against the rye reference sequences of 45S (JF489233) and ω-secalin (AF000227) using a hash size of 10 and an error rate of 20% in the alignment. MosaikCoverage was used to create a graphical view of the representational bias in a base accurate coverage plot. Multiple alignments were allowed, so that a sequence read hitting various regions within the reference shows up multiple times in the coverage distribution. The repeated parts of the reference sequences were visualised by Gepard [74] dotplot generating software.

### Miscellaneous non-coding elements

After identifying organellar, repeat and gene space-related sequence reads, the remaining reads flagged previously as putative repeats and subsequently not identified otherwise were moved to the “Putative repeat” category. The remaining sequence reads were tested as to whether they are related to any type of genomic elements present in *Brachypodium distachyon* 1 k upstream and 1 k downstream, as well as Oryza 1 k upstream and Oryza intergenic or NT Cereal databases using BLASTn.

### Identification of novel repeat elements

Novel repeat elements were identified in two steps by the RepeatScout software suite analysing the sequence reads not tagged in the previous steps. In the initial step using standard settings putative novel repeat element sequences were generated by the software followed by the removal of the low complexity and tandem repeat elements. The derived putative novel repeat elements were verified by realigning them to the sequence reads by RepeatMasker set to at least 50% overlap and 85% similarity. Putative repeat elements hit by more than 10 sequence reads were identified as novel repeat elements. BLASTClust (60% overlap and 90% identity) analysis was used to eliminate putative novel repeats representing the same repeat sequence.

All Roche 454FLX sequence reads generated in this study were submitted to the NCBI GenBank short read archive under the accession number SRA012605.

The database (MSExcel2007, MySQL 5.1 Dump, Unix Text and Windows Text formats) containing the tagging of the sequence reads is available to download at the website: http://www.picme.at/index.php/downloads.

## Supporting Information

Figure S1 **Distribution of sequence reads in the ω-secalin and 45S rDNA loci.**
(TIF)

Table S1 **Statistics of sequence reads and their tagging by different sequence databases.**
(XLS)

Table S2 **List of sequence databases used.**
(XLS)

Table S3 **Annotation of the 1RS gene space related sequence reads.**
(XLS)

Table S4 **SSR region based putative marker sites.**
(XLS)

Table S5 **Classification of the repeat elements of 1RS.**
(XLS)

Table S6 **1RS and 1HS specifc homolog rice loci.**
(XLS)
