# Supplementary figures and images for: Sequence Composition and Gene Content of the Short Arm of Rye (Secale cereale) Chromosome 1
Source: PLoS One. 2012 Feb 6;7(2):e30784. doi: 10.1371/journal.pone.0030784 (PMC3273464; doi:10.1371/journal.pone.0030784)

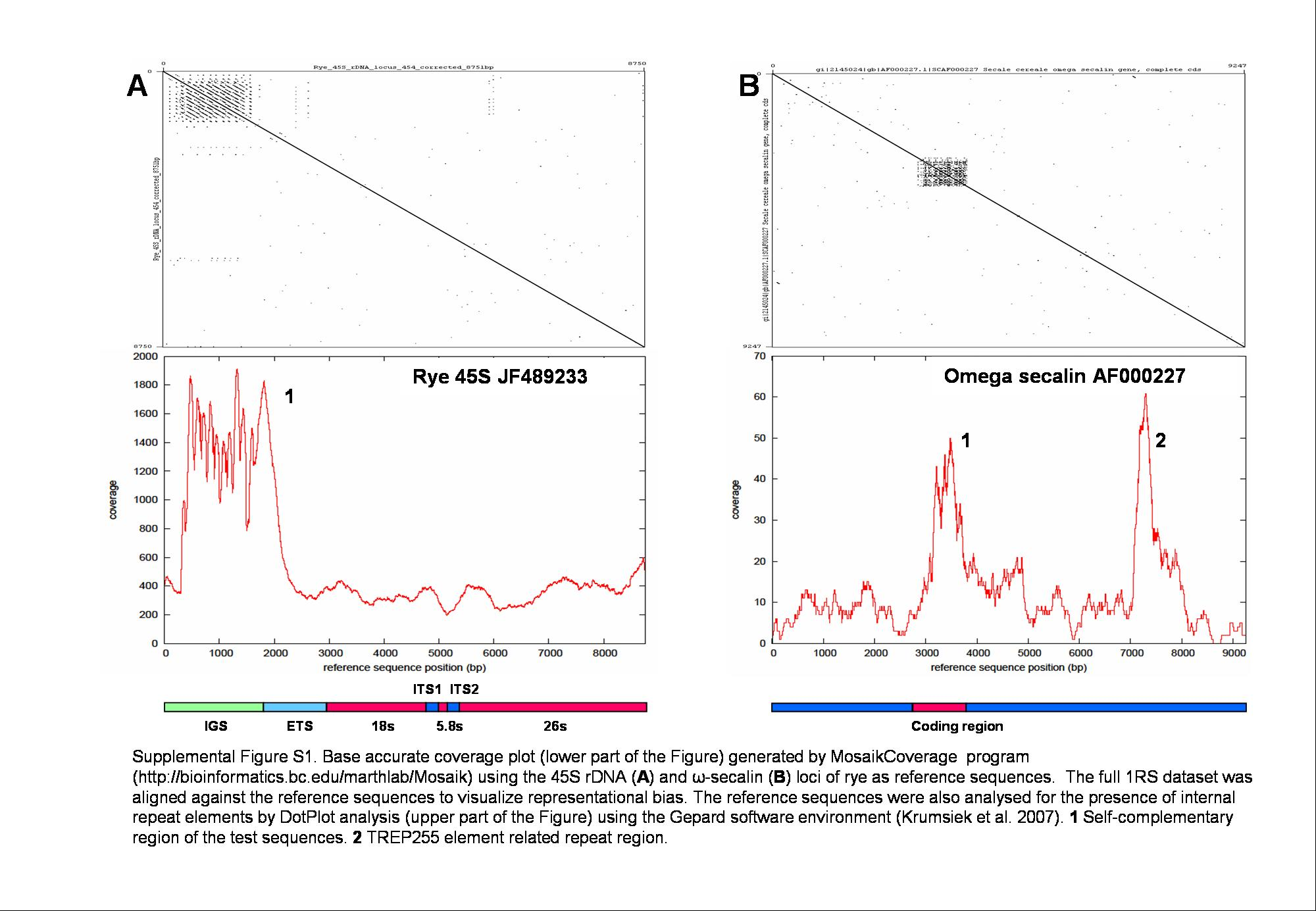

Supplement: Figure S1 — Distribution of sequence reads in the ω-secalin and 45S rDNA loci. (TIF) [file pone.0030784.s001.tif]
